# Rabbit serum albumin as a novel biochemical modulator for enhancing female offspring production in commercial pig breeding through artificial insemination

**DOI:** 10.14202/vetworld.2025.3731-3744

**Published:** 2025-12-09

**Authors:** Thatawat Yodrug, Orachun Hayakijkosol, Tuempong Wongtawan

**Affiliations:** 1 Animal Innovation Research Group, Akkhraratchakumari Veterinary College, Walailak University, Nakhon Si Thammarat 80160, Thailand; 2 Veterinary Preclinical Sciences, College of Science and Engineering, James Cook University, Queensland, 4811 Australia; 3 Center for One Health, Walailak University, Nakhon Si Thammarat 80160, Thailand

**Keywords:** artificial insemination, boar semen, female offspring, rabbit serum albumin, sex ratio, swine breeding

## Abstract

**Background and Aim:**

An increased proportion of female piglets is desirable in commercial swine breeding to improve productivity, facilitate genetic selection, and reduce the need for male castration. However, currently available sex-selection techniques, such as flow cytometry, are costly and impractical for routine field use. This study evaluated the potential of rabbit serum albumin (RSA) as a low-cost biochemical modulator to influence the proportion of female offspring, comparing its effects with those of other albumin sources and determining optimal supplementation conditions for boar semen used for artificial insemination (AI).

**Materials and Methods:**

Eight Landrace boars were initially screened *in vitro* to assess sperm quality and the proportion of X- and Y-bearing sperm following incubation with albumin. Four boars (A, B, E, and G) showing a higher X-sperm proportion were subsequently selected for *in vivo* trials involving 130 sows. Semen was diluted in a conventional extender supplemented with albumin (RSA, porcine serum albumin, or bovine serum albumin) or left unsupplemented (control). The effects of albumin source, concentration (0.1–0.2 mg/mL), incubation temperature (25°C vs. 37°C), duration (5–15 min), and boar variation were examined. Offspring sex ratio and litter size were analyzed using the Kruskal–Wallis test, followed by Dwass–Steel–Critchlow–Fligner pairwise comparisons (p < 0.05).

**Results:**

All albumin treatments significantly increased (p < 0.05) the proportion of female piglets compared with controls. RSA yielded the greatest effect, particularly at 0.1 mg/mL incubated at 37°C for 15 min, producing up to 61.8% female offspring compared with 24.8% in controls. Boars with an initial male-biased sex ratio showed the largest improvement after RSA treatment. Although litter size decreased slightly with albumin supplementation, the difference was not statistically significant (p ≥ 0.05).

**Conclusion:**

Supplementation of semen extenders with RSA effectively increased the proportion of female piglets without compromising fertility. This method offers a practical, scalable, and economical alternative to conventional sex-sorting technologies for swine breeding. Further optimization and larger-scale validation are warranted to ensure consistent litter performance and broader adoption in commercial production systems.

## INTRODUCTION

In commercial swine production, particularly within breeding operations, female pigs are highly valued because they directly contribute to herd replacement and reproductive output, whereas the demand for males has declined due to the widespread use of semen collection and artificial insemination (AI) [1, 2]. Consequently, increasing the proportion of female piglets can substantially enhance economic efficiency, as female breeders accelerate genetic improvement and overall productivity gains [3]. In pig fattening systems, a higher female sex ratio also offers significant welfare and economic benefits by reducing the need for surgical castration of males [4]. Eliminating castration not only minimizes risks of infection, mortality, and labor costs but also prevents the development of boar taint, improving meat quality [5].

Among the currently available techniques, flow cytometry-based sperm sorting remains the most precise method for enriching X-bearing sperm, producing up to 90% female offspring [6, 7]. However, this approach is expensive, technically demanding, and often results in decreased fertility, limiting its use under field or commercial conditions [1]. Alternative immunological methods have been developed to distinguish X- and Y-bearing sperm by targeting variations in surface protein expression [8, 9]. These techniques employ antibodies that bind selectively to sperm antigens, thereby impairing the motility and viability of targeted cells [10–12]. Although less invasive and more affordable than flow cytometry [13–15], immunological separation methods remain inconsistent. For instance, while some studies achieved notable success, reporting up to 74% female calves using antibody-sorted semen without affecting pregnancy rates [15], other studies have shown variable results, and no antibody-based system has achieved consistent reliability in livestock reproduction [9, 16].

Albumin, a major plasma protein, has also been employed in sperm sex-selection techniques, most notably through Ericsson’s albumin gradient method used in human fertility clinics, which modestly increased the proportion of female offspring to 28% [17]. This approach separates X- and Y-bearing sperm based on their differential density, motility, and surface charge interactions with albumin gradients [18]. Mechanistically, albumin-binding to sperm membranes can induce cholesterol efflux, initiating ion fluxes and intracellular signaling cascades that influence sperm motility and viability [19–21]. Because X- and Y-sperm differ in membrane composition and electrochemical properties [22, 23], these interactions may differentially affect their physiological behavior. Despite its promise, the efficiency of albumin-based separation varies widely among species and depends on factors such as albumin source, concentration, and modification techniques. Most existing studies [17, 18, 24–27] have been conducted under *in vitro* conditions, with limited *in vivo* validation, particularly under commercial production environments.

The variability in reported outcomes may be partly attributed to the use of albumins from different species, such as bovine serum albumin (BSA) [28] or human serum albumin [17], each possessing unique structural and biochemical characteristics [29, 30] that influence sperm interaction. Our preliminary investigation demonstrated that rabbit serum effectively promotes the separation of X- and Y-bearing boar sperm *in vitro*, suggesting that rabbit serum albumin (RSA) may be the active component responsible for this effect [31, 32]. Structurally, RSA possesses a distinct ligand-binding pocket compared with other albumins, potentially inducing unique alterations in sperm plasma membranes [33, 34]. These findings indicate that RSA may possess species-specific biochemical properties that modulate sperm function and influence offspring sex ratios.

Despite extensive efforts to manipulate the sex ratio in livestock, most available sex-selection technologies, such as flow cytometry and immunological sperm separation, remain impractical for routine application in the pig industry due to high operational costs, reduced conception rates, and limited scalability under farm conditions. Furthermore, although albumin-based separation methods have been used for decades in humans and some animal species, their efficacy in pigs remains poorly defined. Existing studies are largely confined to *in vitro* experiments, and their findings are inconsistent, often influenced by the albumin source, concentration, and incubation conditions. Specifically, little is known about how species-specific albumins interact with boar spermatozoa to differentially influence X- and Y-bearing sperm physiology. RSA, which exhibits a distinct structural conformation and ligand-binding capacity compared with bovine or porcine serum albumins (PSAs), has not been comprehensively studied for its reproductive effects in swine. Preliminary observations suggest that RSA may alter sperm membrane dynamics, potentially influencing sex-sorting outcomes, yet no systematic *in vivo* investigation under commercial production settings has confirmed this hypothesis. Therefore, a critical knowledge gap persists regarding whether RSA supplementation in semen extenders can reliably and economically bias offspring sex ratio toward females without compromising fertility or litter size.

This study was designed to evaluate the potential of RSA as a biochemical modulator for increasing the proportion of female offspring in pigs under practical breeding conditions. Specifically, the objectives were:

1. To compare the effects of albumin derived from different species, RSA, PSA, and BSA, on offspring sex ratio and total litter size following AI.
2. To determine the optimal concentration, incubation temperature, and duration of RSA treatment that yield the highest proportion of female piglets without adversely affecting fertility.
3. To assess the influence of individual boar variation on the efficacy of RSA-mediated sex ratio modulation.
4. By addressing these objectives through both *in vitro* and *in vivo* evaluations on a commercial pig farm, this research aimed to establish a low-cost, field-applicable, and non-invasive approach to enhance female offspring production, providing an innovative alternative to conventional sex-sorting technologies in the swine industry.

## MATERIALS AND METHODS

### Ethical approval

All animal procedures in this study were conducted in strict accordance with national and institutional animal-care regulations. The complete experimental protocol, including semen collection, handling of boars and sows, housing conditions, and AI procedures, was reviewed and approved by the Animal Care and Use Committee of Walailak University (Approval No. WU-ACUC-67030). The study adhered to the ethical principles outlined in the Walailak University Animal Use Policy, the National Research Council of Thailand (NRCT) guidelines, and international standards, including the Animal Research: Reporting of *In Vivo* Experiments (ARRIVE) 2.0 guidelines, the Directive 2010/63/EU on the protection of animals used for scientific purposes, and the World Organization for Anima Health (formerly OIE) Terrestrial Animal Health Code for the welfare of pigs used in research and breeding.

Prior to study initiation, written permission and cooperation were obtained from the management of the commercial swine farm where in vivo trials were performed. The farm provided confirmation that all animals were healthy, free from major reproductive diseases, and maintained under standard commercial husbandry and biosecurity protocols. No experimental procedures involved invasive surgical intervention, pain, or distress beyond normal farm practices. Semen collection was performed using the standard gloved-hand technique by trained technicians, a procedure considered minimally invasive and routinely used in pig breeding. AI followed established industry practices, and all sows were monitored closely for signs of distress, abnormal behavior, or adverse reactions.

The number of boars (n = 8 for *in vitro* screening; 4 selected for *in vivo* trials) and sows (n = 130) was predetermined based on farm capacity, previous reproductive performance records, and the minimum number required to detect meaningful differences in offspring sex ratio using non-parametric statistics. This ensured that the study would achieve adequate analytical power while minimizing the use of animals. No animals were euthanized or removed from production as a result of this study, and all sows remained in regular farm production cycles after farrowing.

All biosafety procedures, including the use of sterile equipment, pathogen-free extenders, and controlled-temperature semen handling, were implemented to protect both animal health and personnel. Any unexpected clinical signs were recorded and reported to the attending veterinarian; however, no significant welfare-related incidents occurred during the study.

Overall, the research team ensured that animal handling, sampling, and reproductive interventions were performed responsibly and humanely, with continuous oversight by veterinarians and farm personnel trained in animal welfare.

### Study period and location

This study was conducted from September 2024 to May 2025 on a commercial breeding pig farm located in southern Thailand.

### Animals

Eight healthy Landrace boars (1–2 years old) were selected for semen collection. Before the experiment, semen from these boars was routinely collected once per week, and each ejaculate was evaluated to ensure sperm motility and normal morphology exceeding 70%. A total of 130 Landrace sows, with parity ranging from three to five, were enrolled for AI. All animals were maintained in a closed-housing system with strict biosecurity measures to minimize infectious risks. Environmental conditions were controlled at 25°C with a 12-h light/dark cycle throughout the study.

### Preparation of albumin and semen extender

The semen extender used in this experiment was Androstar Plus without antibiotics (Minitube, Tiefenbach, Germany; cat. no. 13531/5001). One pack of extender powder was dissolved in 1 L of sterile distilled water. Three types of highly purified (≥98%) and globulin-free albumin were used (RSA; cat. no. A0764), (PSA; cat. no. A4414), and (BSA; cat. no. A8806) (Sigma-Aldrich Corporation, St. Louis, MO, USA). All extender preparations were carried out under aseptic conditions by trained technicians wearing sterile gloves in a clean laboratory environment.

Albumin was initially dissolved in the extender to a stock concentration of 200 mg/mL, gently mixed, aliquoted into 1 mL microcentrifuge tubes, and stored at −20°C until use. Before semen dilution, aliquots were thawed at 37°C and diluted into 1 L of semen extender to achieve final concentrations of 0.1 mg/mL or 0.2 mg/mL. These concentrations were selected based on the optimal RSA range identified by Korchunjit *et al*. [16].

### Boar semen collection

Semen was collected from eight boars using the gloved-hand technique, and only the gel-free fraction was retained for analysis and insemination. Initial semen quality was assessed on-farm using a phase-contrast microscope (100× and 400×; Nikon, Tokyo, Japan) at 37°C to evaluate sperm motility, and sperm morphology was determined with William’s staining [35]. Only ejaculates with motility >70% and normal morphology >80% were used. Semen was then diluted in the prepared albumin-supplemented extender and stored at 18°C in a semen refrigerator (Minitube) for a maximum of 3 days before AI. Motility was reassessed before insemination, and only samples maintaining >70% motility were utilized.

### Experimental design

**Figure 1:**
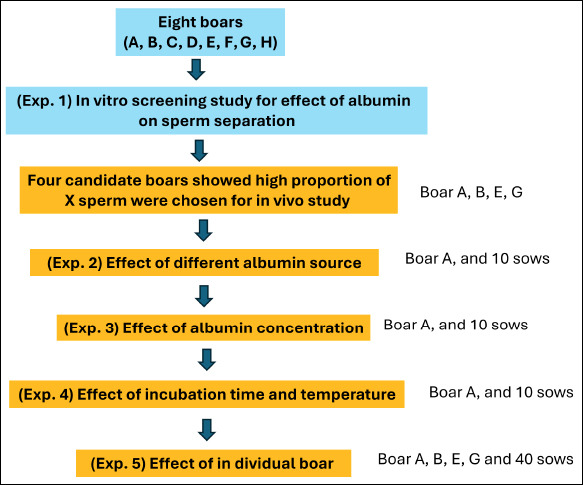
The experimental design of the study. Eight boars were examined *in vitro* to assess the effect of albumin on sperm separation. Four boars with a high proportion of X-bearing sperm were then selected for four *in vivo* artificial insemination experiments that tested the effects of different albumin sources, concentrations, incubation conditions, and individual boars.

The experimental layout is illustrated in Figure 1. Initially, semen from all eight boars was examined *in vitro* to evaluate the influence of albumin on X- and Y-sperm separation. Four boars exhibiting higher proportions of X-bearing sperm (A, B, E, and G) were subsequently selected for *in vivo* trials involving 130 Landrace sows (10 sows per group; total = 13 groups). These experiments assessed the effects of (1) albumin source, (2) albumin concentration, (3) incubation temperature and duration, and (4) individual boar variability.

### *In vitro* assessment of albumin effect

Semen from eight boars was evaluated *in vitro* to identify potential candidates for AI. Diluted semen (500 µL) was mixed with RSA buffer in a 1:1 ratio and incubated at 37°C for 15 min in 1.5 mL microtubes. Control samples were incubated with conventional Androstar® Plus extender alone. Following incubation, the swim-up technique was used to allow motile sperm to migrate toward the surface, facilitating the separation of viable and motile cells from immotile sperm. The albumin incubation step was expected to differentially influence X- and Y-bearing sperm [17, 36]. After 15 min, 100 µL of the upper sperm layer was collected for quality assessment and determination of Y-sperm proportion. Each assay was performed in triplicate per boar.

Sperm motility and morphology were analyzed using a computer-assisted sperm analysis (CASA) system (IVOS 2, Hamilton Thorne, MA, USA) following the manufacturer’s standard protocol for porcine semen. Acrosome integrity was evaluated using fluorescein isothiocyanate-conjugated peanut agglutinin (cat. no. L7381) and ethidium homodimer-1 (cat. no. 46043) (Sigma-Aldrich) under a Nikon Eclipse Ti fluorescence microscope (Nikon Corporation) according to Kaeoket *et al*. [37]. Three replicate slides were examined for each sample.

The proportion of Y-bearing sperm was determined by single-cell real-time polymerase chain reaction (PCR) following Korchunjit *et al*. [38]. From each semen sample (three tubes per boar), 100 individual spermatozoa were isolated for PCR analysis. Two primer sets were used: (i) chromosome 1 primers (positive control; 244 bp product, forward 5′-TTGCACTTTCACGGACGCAGC-3′, reverse 5′-CTAGCCCATTGCTCGCCATAGC-3′) and (ii) Y-chromosome-specific primers (377 bp product, forward 5′-AATCCACCATACCTCATGGACC-3′, reverse 5′-TTTCTCCTGTATCCTCCT GC-3′). Reactions were run using KAPA SYBR® Fast quantitative PCR Master Mix Universal (KAPA Biosystems, Woburn, MA, USA; cat. no. KK4600) on a Rotor-Gene Q system (Qiagen, Hilden, Germany). Data acquisition and melt-curve analysis were performed using Rotor-Gene Q Software (Qiagen, Hilden, Germany). DNase-free water served as a negative control.

### *In vivo* evaluation of albumin effects

**Figure 2:**
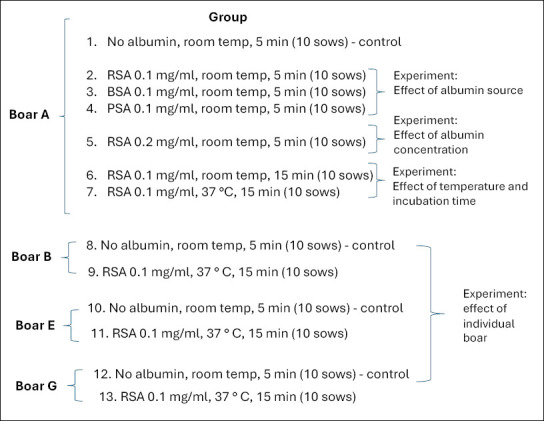
Flowchart of the in vivo studies. Group 1 was the control group. Semen from boar A was used to study the source of albumin (AI in sows, groups 1-4), albumin concentration (groups 1-2 and 5), incubation times (groups 1-2 and 6), and incubation temperatures (groups 1-2 and 7). Semen from boars A to D was used to study the effects of individual boars (groups 1-2 and 8-13).

Based on *in vitro* findings, four boars (A, B, C, and D) showing a high proportion of X-sperm were used for AI of 130 sows. Thirteen experimental groups were created (10 sows per group): group 1 (control), groups 2–4 (albumin source comparison), group 5 (RSA concentration), groups 6–7 (incubation time and temperature), and groups 8–13 (individual boar variation). Figure 2 summarizes the *in vivo* design.

### AI protocol

AI was performed by three experienced technicians using natural estrus detection through the standing reflex. Each sow was inseminated twice, at 24 h and 36 h after the onset of standing heat, using intrauterine catheters (IMV, L’Aigle, France). All procedures were conducted under blinded and randomized conditions. Each insemination dose contained 2 × 109 spermatozoa diluted in 50 mL extender. The total number of piglets born and their sex were recorded immediately after parturition and verified by anatomical inspection.

### Control group (no albumin supplementation)

Semen diluted in conventional extender without albumin served as the control. Samples were maintained at room temperature (25°C–30°C) for 5 min before AI. The control groups included groups 1, 8, 10, and 12.

### Boars used for AI

Figures 1 and 2 illustrate boar usage across experiments. Semen from boar A was used to study albumin source, concentration, incubation duration, and temperature effects (groups 1–7). Semen from boars A–D was used to examine individual boar variation (groups 1, 2, and 8–13) in litter size and offspring sex ratio.

### Albumin from different sources

Semen from boar A was used to evaluate three albumin sources (0.1 mg/mL each): RSA, PSA, and BSA (groups 2–4). Results were compared with the unsupplemented control (group 1).

### Concentration of RSA

Semen from boar A was also used to assess RSA at 0.1 mg/mL (group 2) and 0.2 mg/mL (group 5) relative to the control (group 1).

### Incubation time and temperature

To determine optimal incubation conditions, semen from boar A was treated with 0.1 mg/mL RSA under three regimens: 25°C–30°C (room temperature) for 5 min (group 2), room temperature for 15 min (group 6), and 37°C for 15 min (group 7). All treatments were compared with the control (group 1).

### Individual boar effect

Semen from four boars (A–D) was used to test individual variation. Under the optimized RSA condition (0.1 mg/mL at 37°C for 15 min), each boar’s semen served as its own control (untreated) and treatment. Boar A = groups 1 and 7; B = groups 8 and 9; C = groups 10 and 11; D = groups 12 and 13.

### Data management and statistical analysis

CASA and PCR data were exported to PDF format and tabulated in Google Sheets (Google LLC, CA, USA). Sow identification numbers, pregnancy and farrowing records, litter size, and offspring sex were documented manually and digitized for analysis. Sperm quality parameters, total number of live piglets, number of live female piglets, and percentage of female offspring were expressed as mean ± standard deviation. Data normality was tested using the Shapiro–Wilk test and found to be non-normal. Therefore, comparisons among treatment groups (albumin source, concentration, incubation temperature, and duration) were performed using the non-parametric Kruskal–Wallis (KW) test, followed by Dwass–Steel–Critchlow–Fligner (DSCF) pairwise comparisons. Statistical analyses were conducted using Jamovi software (version 2.6.13; https://www.jamovi.org/) [39]. A p < 0.05 was considered statistically significant.

## RESULTS

### *In vitro* screening for albumin-induced alteration in sperm sex ratio

**Figure 3:**
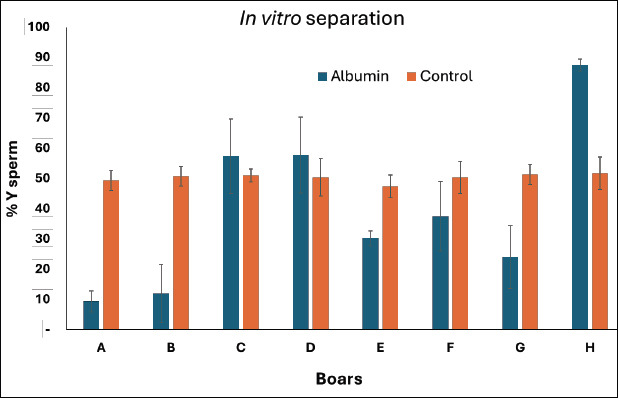
*In vitro* separation of X- and Y-bearing sperm was conducted in eight boars using a semen extender supplemented with rabbit serum albumins. Each boar was tested with rabbit serum albumin. Each bar represents the mean percentage of Y-bearing sperm ± standard deviation.

The outcomes of the *in vitro* screening using semen from eight boars are presented in Figure 3. In the control semen extender, the percentage of Y-bearing sperm ranged between 47.33% and 51.67%, whereas in albumin-treated semen, this range broadened to 9.33%–87.53%. Among all boars, Boar A exhibited the lowest Y-sperm proportion (9.33% ± 3.43%), followed by Boar G (24.00% ± 10.42%), Boar E (30.19% ± 2.47%), and Boar F (37.49% ± 11.48%), with statistically significant differences among individuals (p < 0.05).

Based on these results, boars A, B, E, and G, which showed a higher relative proportion of X-bearing sperm after albumin exposure, were selected for subsequent *in vivo* trials.

### Effect of albumin on sperm quality parameters

**Figure 4:**
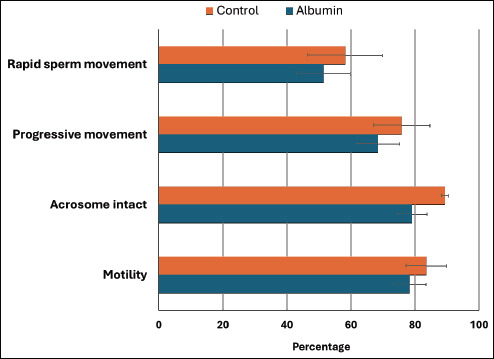
*In vitro* assessment of sperm quality in eight boars before and after supplementation with rabbit serum albumin.

Sperm quality assessment before and after incubation with albumin revealed slight reductions in several motility and integrity parameters, including total motility (83.55% ± 6.29% vs. 78.33% ± 5.17%), rapid sperm movement (58.15 ± 11.62 vs. 51.40 ± 8.46), progressive motility (75.81 ± 8.82 vs. 68.41 ± 6.76), and acrosome integrity (89.33% ± 1.15% vs. 79.08% ± 4.67%). However, none of these differences were statistically significant (p ≥ 0.05) (Figure 4). These findings indicate that albumin supplementation did not adversely affect boar sperm viability or morphology under the tested conditions.

### Effect of albumin source on offspring sex ratio

The farrowing rate in this experiment was 100% across all treatment groups. As shown in Table 1, the total number of live piglets tended to be slightly higher in the control group compared to albumin-supplemented groups, although the difference was only borderline significant (KW p = 0.02; DSCF p ≥ 0.08). In contrast, the number and proportion of female piglets were significantly higher in all albumin-treated groups (p < 0.001) than in the control. Among albumin types, RSA produced the highest number (8.50 ± 1.35) and percentage of female piglets (55.63% ± 7.52%), both significantly greater (p < 0.001) than the control (24.76% ± 0.05%). No significant differences were detected between RSA, PSA, and BSA treatments (p ≥ 0.80).

**Table 1:** The total number of live piglets and female piglets resulting from artificial insemination using semen extender supplemented with RSA, PSA, or BSA. This study used semen from boar A. The piglet’s value is represented as mean ± standard deviation.

Albumin sources	Number of Inseminated sows	Number of farmed sows	Total number of live piglets	Total number of live female piglets	

				Number	Percentage
Control (no albumin)	10	10	18.70 ± 5.70^a^	4.50 ± 1.08^a^	24.76 ± 0.05^a^
RSA 0.1 mg/mL	10	9	15.40 ± 2.37^a^	8.50 ± 1.35^b^	55.63 ± 7.52^b^
PSA 0.1 mg/mL	10	9	14.20 ± 2.15^a^	7.00 ± 1.25^b^	50.08 ± 0.09^b^
BSA 0.1 mg/mL	10	10	14.90 ± 1.37^a^	7.50 ± 1.27^b^	50.45 ± 0.50^b^
*p-value			0.02	<0.0001*	<0.0001*
Effect size			0.23	0.61	0.57

^a,b^Different superscripts denote a significant difference (p < 0.05) in the same column analyzed by Dwass–Steel–Critchlow–Fligner pairwise comparisons.

* p-value of the Kruskal–Wallis test, p < 0.05 represents a significant difference. RSA = Rabbit serum albumin, PSA = Porcine serum albumin, BSA = Bovine serum albumin.

### Effect of RSA concentration on offspring sex ratio

Table 2 summarizes the influence of RSA concentration on offspring sex distribution. Although the total number of live piglets in both RSA-treated groups (0.1 mg/mL and 0.2 mg/mL) tended to be lower than the control, the differences were not statistically significant (p = 0.40). In contrast, the number and percentage of female piglets were markedly higher in both RSA-treated groups compared with the control (p < 0.001). Between the two concentrations, no significant difference was detected (p ≥ 0.05), though the 0.1 mg/mL RSA treatment yielded the highest proportion of female piglets (55.63% ± 7.52%).

**Table 2:** Effect of rabbit serum albumin supplementation on the total number of live and female piglets. This study used semen from boar A. The piglet’s value is represented as mean ± standard deviation.

Albumin concentration	Number of Inseminated sows	Number of farmed sows	Total number of live piglets	Total number of live female piglets	

				Number	Percentage
Control (no albumin)	10	10	18.70 ± 5.70^a^	4.50 ± 1.08^a^	24.76 ± 0.05^a^
RSA, 0.1 mg/mL	10	9	15.40 ± 2.37^a^	8.50 ± 1.35^b^	55.63 ± 7.52^b^
RSA, 0.2 mg/mL	10	10	15.70 ± 2.75^a^	7.70 ± 1.34^b^	50.02 ± 10.12^b^
*p-value			0.40	<0.0001*	<0.0001*
Effect size			0.06	0.69	0.69

^a,b^Different superscripts denote a significant difference in the same column (p < 0.01) analyzed by Dwass–Steel–Critchlow–Fligner pairwise comparisons.

* p-value of the Kruskal–Wallis test, p < 0.05 represents a significant difference. RSA = Rabbit serum albumin.

### Effect of incubation temperature and duration on offspring sex ratio

The total number of live piglets tended to be lower in RSA-treated semen, irrespective of incubation temperature or time, though this reduction was not significant (p = 0.27) (Table 3). All RSA treatment conditions, however, significantly increased the number and percentage of female piglets relative to the control (p < 0.05). The highest proportion of female offspring (61.80% ± 0.12%) was achieved when semen supplemented with 0.1 mg/mL RSA was incubated at 37°C for 15 min, though this difference was not statistically significant compared with room temperature treatments (p ≥ 0.05).

**Table 3:** Effect of incubation times and temperatures of RSA supplementation in semen extender on the total number of live and female piglets. This study used semen from boar A. The piglet’s value is represented as mean ± standard deviation.

Incubation conditions	Number of Inseminated sows	Number of farmed sows	Total number of live piglets	Total number of live female piglets	

				Number	Percentage
Control (no albumin) Room temp. 5 min	10	10	18.70 ± 5.70	4.50 ± 1.08	24.76 ± 0.05^a^
RSA (0.1 mg/mL) Room temp. 5 min	10	9	15.40 ± 2.37	8.50 ± 1.35	55.63 ± 7.52^b^
RSA (0.1 mg/mL) Room temp. 15 min	10	9	16.60 ± 1.65	9.60 ± 2.72	57.52 ± 0.14^b^
RSA (0.1 mg/mL) 37°C 15 min	10	10	14.80 ± 2.94	9.10 ± 2.13	61.80 ± 0.12^c^
*p-value			0.27	0.04	0.0004*
Effect size			0.09	0.19	0.47

^a-c^Different superscripts denote a significant difference (p < 0.05) in the same column (control vs. treatments) analyzed by Dwass–Steel–Critchlow–Fligner pairwise comparisons. The room temperature was approximately 25°C–30°C.

* p-value of the Kruskal–Wallis test, p < 0.05 represents a significant difference. RSA = Rabbit serum albumin.

### Effect of individual boar variation on offspring sex ratio

A distinct sex ratio bias was evident among the boars (Table 4). Boars A and B produced a higher proportion of male offspring in the control groups, with corresponding female proportions of 24.76% ± 0.05% and 32.13% ± 0.07%, respectively. In contrast, Boar E displayed a nearly balanced sex ratio (42.07% ± 0.06% female), while Boar G showed a slight natural bias toward female offspring (51.76% ± 0.20%).

**Table 4:** Effect of individual boars on the total number of live and female piglets. Treatment group: Semen was supplemented with 0.1 mg/mL RSA and incubated at 37°C for 15 min. Each semen sample was diluted with a conventional semen extender (control group, without albumin) and incubated at room temperature (25°C–30°C) for 5 min. The piglet’s value is represented as mean ± standard deviation.

Boars	Groups	Number of inseminated sows	Number of farmed sows	Total number of live piglets	Number of live female piglets	

					Number	Percentage
A	Control	10	10	18.70 ± 5.70	4.50 ± 1.08	24.76 ± 0.05
	RSA	10	9	14.80 ± 2.94	9.10 ± 2.13	61.80 ± 0.12
	Fold change			−1.26	+2.02	+2.50
	p-value			0.07	<0.0001*	<0.0001*
B	Control	10	9	19.70 ± 3.74	6.30 ± 1.57	32.13 ± 0.07
	RSA	10	10	14.90 ± 2.47	9.00 ± 2.26	60.34 ± 0.11
	Fold change			−1.32	+1.42	+1.87
	p-value			0.008*	0.005*	0.0002*
E	Control	10	9	17.40 ± 3.08	7.40 ± 1.49	42.07 ± 0.06
	RSA	10	10	14.80 ± 2.78	10.00 ± 2.54	67.00 ± 0.13
	Fold change			−0.85	+1.35	+1.59
	p-value			0.03*	0.002*	0.0002*
G	Control	10	9	18.30 ± 2.50	8.80 ± 2.65	51.76 ± 0.20
	RSA	10	10	14.70 ± 2.79	10.10 ± 2.69	68.00 ± 0.09
	Fold change			−1.24	+1.15	+1.31
	p-value			0.05	0.02*	<0.0001*

* A significant difference (p < 0.05) between the control and treatment groups in each boar analyzed by Mann–Whitney U-test. RSA = Rabbit serum albumin.

After RSA supplementation (0.1 mg/mL at 37°C for 15 min), the number and percentage of female piglets significantly increased across all boars (p < 0.03), with the greatest improvements observed in Boars A and B, which initially produced male-biased litters. However, a mild decline in total litter size was noted across RSA-treated groups, reaching statistical significance in Boars A and B (p < 0.05).

## DISCUSSION

### Boar-associated sex ratio bias and its implications

A notable observation from this study was a sex ratio bias among individual boars, highlighting the inherent male-line influence on offspring sex. This bias also emphasized the positive role of albumin, particularly RSA, in counterbalancing male-dominant tendencies and increasing the proportion of female piglets. Sex-biased offspring production has been reported in wild boars and is often attributed to paternal rather than maternal factors [40, 41]. However, data on this phenomenon in farmed pigs remain limited, and the determinants of such bias are poorly understood [42, 43]. According to farm records, Landrace boars exhibited a higher incidence of sex-biased litters compared with other breeds (unpublished observations). This suggests a possible genetic component underlying the bias. Future studies should include proteomic profiling of seminal plasma and genetic marker analyses to elucidate the molecular and heritable basis of sex ratio bias in boars.

### Mechanistic insights: albumin-mediated modulation of sperm physiology

The current findings demonstrated that albumin, particularly RSA, significantly increased the proportion of X-bearing sperm *in vitro* and raised the number of female piglets after AI. These results align with findings of Korchunjit *et al*. [36] showing that albumin-rich media enhance X-sperm survival following incubation or cryopreservation of boar semen. Albumin’s ability to bind to sperm plasma membranes induces cholesterol efflux and alterations in ion flux, initiating cascades associated with capacitation, the acrosome reaction, and motility regulation [19–21].

Because X- and Y-bearing sperm differ in membrane composition, electrochemical potential, and metabolic rate [22, 23, 44–46], albumin may interact differentially with each subpopulation. The superiority of RSA over other albumins (PSA and BSA) may stem from its unique structural conformation, featuring a distinct ligand-binding pocket and altered lipid affinity [33, 34]. These structural attributes facilitate enhanced cholesterol efflux and membrane fluidity, promoting selective destabilization of Y-sperm membranes and increasing the relative proportion of viable X-sperm. Thus, RSA’s species-specific biochemical profile may underlie its greater efficiency as a bioselective modulator in sperm sex-sorting.

### Effect of RSA concentration and physiological responses

Increasing RSA concentration was associated with fewer female piglets, suggesting that concentration optimization is critical. Elevated albumin concentrations can increase osmolarity, potentially inducing osmotic stress and impairing sperm motility and viability [47, 48]. High albumin levels may also accelerate capacitation through the cellular signaling pathway involved cyclic adenosine monophosphate (cAMP), protein kinase A (PKA) and calcium, triggering premature hyperactivation and excessive adenosine triphosphate (ATP) consumption, an effect known as sperm exhaustion [49, 50]. Consequently, sperm exposed to higher RSA concentrations may exhibit reduced functional longevity, which explains the observed trend toward fewer viable female embryos.

### Practical advantages of RSA-based sperm modulation

This study demonstrated that supplementing semen extenders with RSA, particularly when combined with optimal temperature (37°C) and incubation duration (15 min), represents a low-cost, non-invasive, and scalable approach to biasing offspring sex. RSA supplementation costs are over 100 times lower than flow cytometry or antibody-based sexing techniques, offering substantial economic benefits for resource-limited breeding operations. The method aligns well with the concept of biochemical-assisted sperm sorting [46–51], in which species-specific albumin functions as a bioselective molecular filter. This approach may influence sperm membrane cholesterol efflux, calcium flux, and intracellular signaling to preferentially support X-sperm survival and fertilizing capacity [33, 52, 53].

### Relationship between *in vitro* and *in vivo* outcomes

A consistent pattern was observed between *in vitro* and *in vivo* results. Boars A and B, which exhibited the greatest reductions in Y-sperm proportions during *in vitro* screening, also produced the highest proportions of female offspring following AI. This suggests that *in vitro* sperm screening using PCR and swim-up methods can serve as an efficient pre-selection tool for identifying boars most responsive to albumin treatment. Implementing this screening step before large-scale AI could enhance both the predictability and economic efficiency of sex-preselection programs in commercial pig production.

### Comparison with previous research in other species

In other livestock species, most albumin-based sex-sorting studies have remained limited to *in vitro* trials. Reports in rams and bulls indicate sex-sorting efficiencies between 65% and 70% using albumin gradients [26, 54, 55], values comparable to those achieved in this study. However, few investigations have validated such results under *in vivo* conditions, underscoring the novelty of the current study. Future research should thus focus on field-level validation and optimization of albumin-mediated sex-selection across different breeds and species.

### Limitations and breed-dependent variability

Although RSA effectively increased the proportion of female offspring, outcomes varied among individual boars and breeds. Landrace boars appeared particularly responsive, corroborating earlier findings that genetic and physiological variability influences sperm sex-sorting performance [32, 56]. Boar-specific responses to albumin concentration and source suggest that individual optimization may be necessary for consistent success. To enhance precision, future studies should integrate genomic and proteomic profiling [45, 52, 53, 57] to identify molecular markers linked to albumin-binding affinity, sperm membrane structure, and capacitation response.

### Potential causes of reduced litter size

Although RSA supplementation significantly increased the female sex ratio, a minor reduction in total litter size was observed. This trend may be attributed to accelerated capacitation and early hyperactivation of spermatozoa treated with RSA, leading to shorter motility duration and potential energy depletion [33, 52, 53]. CASA indicated that RSA-treated sperm became hyperactive more rapidly than controls but remained motile for a shorter period. In addition, albumin may induce mild oxidative stress via lipid peroxidation and the generation of reactive oxygen species (ROS) during capacitation [52, 58]. While moderate ROS levels facilitate fertilization, excessive ROS may reduce sperm viability, particularly in Y-sperm, thereby slightly reducing the total number of fertilized ova. Increasing sperm concentration per insemination dose may counterbalance this limitation, but this requires empirical verification.

### Study constraints and practical feasibility

A limitation of this study was the relatively small number of sows per group, due to farm-level constraints and the desire to evaluate multiple variables with limited animals. Despite this, statistically significant trends were observed, confirming the robustness of the data. Conducting the research under commercial farm conditions provided valuable real-world insight into the feasibility of albumin-based interventions in standard pig production systems.

### Future perspectives and research directions

Further studies should aim to optimize this strategy to maximize the proportion of female piglets without reducing litter size. Combining RSA supplementation with other practical interventions, such as acidic extenders that selectively impair Y-sperm viability [59, 60], could enhance efficiency. Molecular docking and proteomic analyses should be employed to identify key sperm receptors and signaling pathways involved in albumin-mediated sex modulation. Moreover, cross-species validation in cattle, goats, and dogs could determine whether RSA’s mechanism of action is conserved across mammals. Integrating RSA-based media with antioxidants or pH modulation may also improve sperm longevity and maintain the desired sex bias.

Finally, the observed boar-dependent variation suggests that individualized RSA formulations could be developed for high-value breeding programs. Although such customization may be impractical for large-scale commercial herds, it holds promise for elite genetic selection, where female offspring and reduced castration costs yield substantial economic benefits. In Thailand, for instance, female breeding pigs are often valued up to 10 times more than their male counterparts, underscoring the transformative potential of this technology for sustainable, profitable swine production.

## CONCLUSION

The present study demonstrated that supplementing boar semen extenders with RSA significantly increased the proportion of female offspring in pigs without compromising fertility or farrowing performance. Optimal conditions, 0.1 mg/mL RSA, incubated at 37°C for 15 min, yielded the highest female sex ratio (≈62%) while maintaining acceptable semen quality. RSA outperformed bovine and porcine albumins, likely owing to its unique molecular structure and stronger affinity for sperm membranes, which facilitates selective modulation of X- and Y-bearing sperm.

This approach offers a simple, low-cost, and field-applicable alternative to flow cytometry or antibody-based sex-sorting systems and could be especially beneficial for small and medium-scale farms seeking to enhance productivity through female-biased litters. The principal strength of the study lies in its combination of controlled *in vitro* screening and commercial-scale *in vivo* validation.

However, responses varied among individual boars, and a slight reduction in litter size was observed, indicating that breed- and boar-specific optimization is required. Future research should integrate proteomic and genomic profiling to predict boar responsiveness, refine extender formulations, and explore synergistic use with acidic or antioxidant-modified media.

Overall, RSA-based semen supplementation represents a promising, scalable, and biologically grounded method for enhancing female offspring production and advancing precision reproductive management in the swine industry.

## DATA AVAILABILITY

The data supporting the findings of this study can be obtained from the corresponding author.

## AUTHORS’ CONTRIBUTIONS

TY: Conceptualization, investigation, acquisition of funding, formal analysis, and writing the original manuscript. TW: Conceptualization, investigation, funding acquisition, formal analysis, project administration, resource allocation, and writing the original manuscript. OH: Conceptualization, supervision, and validation of the data. All authors have read and approved the final version of the manuscript.
